# Recurrent emergent hernia repairs: who is at risk?

**DOI:** 10.1007/s00464-025-11914-y

**Published:** 2025-06-19

**Authors:** Erin E. Isenberg, Joshua Sinamo, Michael A. Rubyan, Annie Ehlers, Dana A. Telem

**Affiliations:** 1 Department of Surgery, University of Texas at Southwestern, Dallas, TX USA; 2 National Clinician Scholars Program, Institute for Healthcare Policy and Innovation, University of Michigan, North Campus Research Complex Building 16, 2800 Plymouth Road, Ann Arbor, MI 48109 USA; 3 Department of Surgery, University of Michigan, Ann Arbor, MI USA; 4 School of Public Health, University of Michigan, Ann Arbor, MI USA

**Keywords:** Hernia, Medicare, Disparities

## Abstract

**Background:**

Patients undergoing recurrent emergent hernia repairs may represent some of the most vulnerable patients in the healthcare system. However, this population has not been adequately characterized to date, limiting identification of opportunities for intervention.

**Methods:**

We conducted a retrospective cross-sectional study of Medicare beneficiaries who underwent an index emergent or urgent ventral hernia repair between 2011 and 2021. We performed a multivariable risk-adjusted Royston–Parmar survival analysis for cumulative recurrent emergent hernia repair incidence within 10 years, accounting for patient comorbidities, demographics, and repair characteristics. We assessed patient, provider, and community factors associated with an increased risk of recurrent emergent hernia repair. We used the Social Vulnerability Index, a publicly available score from the CDC reflecting 15 sociodemographic factors, to measure social risk.

**Results:**

Among 120,227 Medicare beneficiaries who underwent emergent hernia repair during the study period, the mean (SD) age was 71 (12) and 58% were female. At 10 years, the cumulative incidence of recurrent emergent hernia repair was 6.4% (95% CI 6.2–6.7). Median time to recurrent repair was 1.6 years [IQR 0.8–3.2]. Risk-adjusted factors associated with increased risk for recurrent emergent repair include female sex (10-year Hazard Ratio [HR] 1.58, 95% CI [1.24–2.02]), Hispanic ethnicity (10-year HR 1.19, 95% CI [1.01–1.41]), and patients in the highest quintile of social vulnerability (10-year HR 1.33, 95% CI [1.03–1.71]). Hernias repaired via open approach (10-year HR 1.44, 95% CI [1.03- 2.01) and at for-profit hospitals (10-year HR 1.16, 95% CI [1.06–1.26]) also experienced increased risk.

**Conclusion:**

Female, Hispanic, and socially vulnerable patients are at increased risk of recurrent emergent hernia repairs, as well as patients receiving an open index repair and care at for-profit hospitals. Our study highlights opportunities for intervention, including consideration of index repair approach and populations that may benefit from closer follow-up and earlier elective intervention for hernia recurrence.

**Supplementary Information:**

The online version contains supplementary material available at 10.1007/s00464-025-11914-y.

Surgical conditions that are preference sensitive (i.e. conditions with multiple options for treatment without a scientifically proven “best” option) may represent a unique opportunity to understand critical gaps between well-resourced and vulnerable patients. These conditions, such as abdominal wall hernia repair, should be managed electively with shared decision making. However, this requires adequate access to care, high health literacy, patient-physician trust, financial resources, and the ability to optimize medical comorbidities such as smoking, diabetes, and obesity before being offered surgery [1–4], As a result, patients with social and systemic barriers likely represent the population at highest risk for repeated emergent presentations rather than elective management for these conditions. Up to 20% of hernias are repaired emergently, and nearly 50% for older adults, indicating a major opportunity for improvement [5].

While the need for an initial emergent hernia repair is not always preventable, patients undergoing repeated emergent hernia repairs represent a clear missed opportunity for intervention. The incidence and risk factors of this patient population are poorly characterized. Prior work has focused primarily on clinical risk factors for an initial emergent repair, such as age, comorbidities, and hernia-to-neck ratio, but has not described incidence of recurrent emergent repairs or the individual and systemic social factors that put them at risk [6–8]. Given the up to 15-fold increase in morbidity, mortality, and recurrence associated with emergent hernia repairs, characterizing this population could serve to inform the patients and systems with the highest need for resources and interventions to mitigate these downstream adverse outcomes in preference sensitive conditions [9].

In this context, we designed a study to characterize patient, provider, and community factors associated with an increased risk of recurrent emergent hernia repair among Medicare beneficiaries, evaluating both clinical and social risks. Medicare data affords a unique opportunity to study this question given the comprehensive longitudinal data in the population most affected by emergent hernia surgery. We performed a risk-adjusted survival analysis for cumulative recurrent emergent hernia repair incidence within 10 years.

## Methods

### Data source and patient population

This is a retrospective study using data from the 100% fee-for-service Medicare claims data between 2011 and 2021. We identified and included adult (18+) beneficiaries who underwent an inpatient index urgent or emergent ventral/incisional or umbilical hernia repair using the relevant CPT, diagnosis, and procedure codes from the International Classification of Diseases, Tenth Revision, Clinical Modification (ICD-10-CM) and International Classification of Diseases, Tenth Revision, Procedure Coding System (ICD-10-PCS) (Supplemental Table 1). Patients were excluded from the index cohort if they had a hernia repair within 2 years prior to the start of the study period or if their repair was associated with Current Procedural Terminology (CPT) codes for repair of a recurrent ventral hernia (49,565, 49,566, 49,656, 49,657). Emergent procedures were determined by urgent or emergent admission status as defined by the Claim Inpatient Admission Type Code, validated in prior surgical studies using Medicare claims [10, 11].

Hospital characteristics data were obtained from the American Hospital Association Annual Survey. Each admission was linked to a hospital by a unique hospital identifier and year of operation located in the Medicare Provider Analysis and Review (MEDPAR) file. Social Vulnerability Index (SVI) data were obtained from the Centers for Disease Control and Prevention [12], and linked with each admission on a zip code level based on the beneficiary’s residence and year of operation. We utilized Area Health Resources Files data from the Health Resources and Services Administration to determine the primary care health professional shortage area (PC-HPSA) designation for each admission based on the beneficiary’s county of residence and year of operation [13]. This study was approved by the University of Michigan Investigation Review Board and deemed exempt due to the use of secondary data.

### Exposures

Exposures of interest were categorized as patient, provider, or community-level factors that we hypothesized to be risk factors associated with the need for an additional emergent hernia repair. Patient factors included sex, race/ethnicity, and dual Medicare-Medicaid eligibility. Dual eligibility was chosen as an established proxy for socioeconomic risk in the setting of limited patient-level social determinants of health. Provider-level factors included hernia surgeon volume, repair approach (open vs minimally invasive), and hospital ownership (for-profit vs. non-profit). Hernia surgeon volume was measured by tallying and ranking the total number of hernia surgeries performed by each surgeon throughout the study period and grouping them into tertiles (low, medium, high).

Community-level factors included the SVI and the primary care health professional shortage area (PC-HPSA) designation. SVI comprises 15 social factors divided into 4 subdomains: socioeconomic status, household characteristics, racial and ethnic minority status, and housing type and transportation. We transformed SVI reported at the census tract level into ZIP code level using a population-weighted average within each ZIP code [14]. The score is a percentile ranging from 0 (lowest vulnerability) to 1 (highest vulnerability) and was evaluated as a categorial variable where patients were stratified based on ordinal quintiles, with the quintile score of 0.20 or lower designated as the lowest vulnerability and the quintile score of 0.80 or greater designated as the highest vulnerability. This was done based on previous studies evaluating SVI and surgical outcomes [10, 11].

The Health Resources and Services Administration assigns primary care HPSAs to geographic areas with a shortage of primary care physicians, defined as a population-to-full-time-equivalent primary care physician ratio of at least 3500:1. Designations include non-HPSA, partial-county HPSA (populations with high needs at a subcounty level), or whole-county HPSA (entire county meets the minimum population-to-PCP ratio). Recent evidence has demonstrated that patients in primary care shortage areas experience higher rates of emergency surgery [2].

### Outcome

The primary outcome was recurrent emergent ventral hernia repair, identified throughout the follow-up period of 10 years after the index using the relevant ICD-9 or ICD-10 codes, admission status, and Medicare Beneficiary Identifier uniquely available for each patient.

### Analysis

First, patient, clinical, and hospital characteristics of beneficiaries who did and did not undergo a recurrent emergent hernia repair were compared using *T* and Chi-square tests. As a sensitivity check, we also evaluated the patient, clinical, and hospital characteristics of beneficiaries who underwent a repeat emergent repair with those who underwent a repeat elective repair. Given we could not identify clinical recurrences without an operation, this provided the next closest comparison group that would exclude patients with no recurrence.

We then calculated the median time to recurrent repair, 30-day complications, and mortality. We then performed a multivariable Royston–Parmar flexible parametric survival hazards regression model to evaluate the primary outcome of recurrent emergent ventral hernia repair in the overall cohort. The advantage of the flexible parametric survival regression model is the ability to accommodate and generate absolute measures of effect (i.e. a hazard ratio) for time-varying covariates, a limitation of the Cox proportional hazards regression model [15–17]. The model fits a restricted cubic spline to allow model flexibility for the baseline log cumulative hazard on the proportional hazards scale.

Our covariates included age, sex, race/ethnicity, Elixhauser comorbidities, dual Medicare-Medicaid eligibility, beneficiary Medicare status code (i.e. end-stage renal disease or disability status), surgical approach, hernia location, mesh use, component separation use, hernia surgeon volume, hospital bed size, teaching hospital status, hospital ownership, hospital nurse ratio, PC-HPSA designation, SVI, rural/urban area commuting status of beneficiary residence, and U.S. region of beneficiary residence. Statistical analyses were performed using Stata version 18.5 (StataCorp) by our statistician. STROBE reporting guidelines were followed [18].

## Results

### Patient characteristics

From 2011 to 2021, 120,227 Medicare beneficiaries underwent an emergent ventral hernia repair (Table 1). The mean (SD) age was 71 (12) and 58% were female. Of those undergoing initial emergent repair, 4018 (3.3%) underwent a recurrent emergent repair during the study period. Those undergoing recurrent emergent repair were more likely to be younger (67.4 vs 71.6, p < 0.001), female (67% vs. 58%, p < 0.001), Black (14% vs 12%, p < 0.001) or Hispanic (3.7% vs 2.5%, p < 0.001), dual-eligible (35% vs 28%, p < 0.001), and enrolled in Medicare for disability status (27% vs 17%, p < 0.001) as compared to those not undergoing recurrent emergent repair (Table 1). When comparing the cohort that underwent repeat emergent repair (n = 4018) versus only those that underwent repeat elective repair (n = 8110), results were similar, and the recurrent elective repair group closely reflected characteristics of the cohort that did not undergo a recurrent operation (Supplemental Table 2).

**Table 1:** Patient characteristics

Covariate		All (N = 120,227) % column	Had a recurrent urgent/emergent repair	p-value	
			No (n = 116,209) % column	Yes (n = 4018) % column	
Age	Mean (SD)	71.4 (12.0)	71.6 (12.0)	67.4 (12.5)	< 0.001
Sex	Female	70,072 (58.3%)	67,377 (58.0%)	2695 (67.1%)	< 0.001
	Male	50,155 (41.7%)	48,832 (42.0%)	1323 (32.9%)	
Race	White	99,046 (82.4%)	95,867 (82.5%)	3179 (79.1%)	< 0.001
	Black	13,973 (11.6%)	13,415 (11.5%)	558 (13.9%)	
	Hispanic	3027 (2.5%)	2878 (2.5%)	149 (3.7%)	
	Other races	4181 (3.5%)	4049 (3.5%)	132 (3.3%)	
Repair approach	Open	100,976 (84.0%)	97,539 (83.9%)	3437 (85.5%)	0.007
	MIS	19,251 (16.0%)	18,670 (16.1%)	581 (14.5%)	
Repair location	Incisional-ventral	80,758 (67.2%)	77,557 (66.7%)	3201 (79.7%)	< 0.001
		39,469 (32.8%)	38,652 (33.3%)	817 (20.3%)	
Mesh use		49,616 (41.3%)	47,907 (41.2%)	1709 (42.5%)	0.100
Component separation use		5086 (4.2%)	4916 (4.2%)	170 (4.2%)	1.000
Enterectomy	Umbilical	11,325 (9.4%)	10,846 (9.3%)	479 (11.9%)	< 0.001
Number of elixhauser comorbidities (out of 29)	Zero	5348 (4.4%)	5186 (4.5%)	162 (4.0%)	0.126
	One	13,610 (11.3%)	13,184 (11.3%)	426 (10.6%)	
	Two and above	101,269 (84.2%)	97,839 (84.2%)	3430 (85.4%)	
Hernia surgeon volume [mean: low = 13.8, med = 39.4, high = 110.2]	Low (1 to 25)	40,402 (33.6%)	39,076 (33.6%)	1326 (33.0%)	0.145
	Medium (26 to 56)	39,843 (33.1%)	38,454 (33.1%)	1389 (34.6%)	
		39,982 (33.3%)	38,679 (33.3%)	1303 (32.4%)	
Dual (Medicare-Medicaid) eligible	High (57 +)	33,692 (28.0%)	32,285 (27.8%)	1407 (35.0%)	< 0.001
Beneficiary Medicare status code	Aged with end-stage renal disease (ESRD)	2716 (2.3%)	2614 (2.2%)	102 (2.5%)	< 0.001
	Aged without ESRD	92,978 (77.3%)	90,324 (77.7%)	2654 (66.1%)	
	Disabled with ESRD	2269 (1.9%)	2162 (1.9%)	107 (2.7%)	
	Disabled without ESRD	20,835 (17.3%)	19,748 (17.0%)	1087 (27.1%)	
	ESRD only	1429 (1.2%)	1361 (1.2%)	68 (1.7%)	
Hospital bed size	< 250	46,907 (39.0%)	45,386 (39.1%)	1521 (37.9%)	0.198
	250–499	42,213 (35.1%)	40,798 (35.1%)	1415 (35.2%)	
	500 +	31,107 (25.9%)	30,025 (25.8%)	1082 (26.9%)	
Teaching hospital	Non-teaching	39,303 (32.7%)	37,944 (32.7%)	1359 (33.8%)	0.124
	Teaching	80,924 (67.3%)	78,265 (67.3%)	2659 (66.2%)	
Hospital ownership	For-profit	17,391 (14.5%)	16,746 (14.4%)	645 (16.1%)	0.006
	Not-for-profit	89,947 (74.8%)	87,022 (74.9%)	2925 (72.8%)	
	Other (government)	12,889 (10.7%)	12,441 (10.7%)	448 (11.1%)	
Nurse staffing ratio	Mean (SD)	17.25 (0.0%)	8.68 (3.4)	8.57 (13.67)	0.6157
Health Professional Shortage Area (HPSA)	Non-HPSA	12,614 (10.5%)	12,192 (10.5%)	422 (10.5%)	< 0.001
	Part county	82,754 (68.8%)	80,147 (69.0%)	2607 (64.9%)	
	Whole county	24,859 (20.7%)	23,870 (20.5%)	989 (24.6%)	
Social Vulnerability Index (SVI)	Low	20,317 (16.9%)	19,706 (17.0%)	611 (15.2%)	< 0.001
	Medium–low	21,853 (18.2%)	21,176 (18.2%)	677 (16.8%)	
	Medium	22,995 (19.1%)	22,253 (19.1%)	742 (18.5%)	
	Medium–high	25,725 (21.4%)	24,875 (21.4%)	850 (21.2%)	
	High	29,337 (24.4%)	28,199 (24.3%)	1138 (28.3%)	
Population density, urbanization, and daily commuting	Not-rural	94,472 (78.6%)	91,280 (78.5%)	3192 (79.4%)	0.181
	Rural	25,755 (21.4%)	24,929 (21.5%)	826 (20.6%)	
U.S. Region	South	47,880 (39.8%)	46,262 (39.8%)	1618 (40.3%)	0.025
	Midwest	28,543 (23.7%)	27,650 (23.8%)	893 (22.2%)	
	Northeast	24,787 (20.6%)	23,898 (20.6%)	889 (22.1%)	
	West	19,017 (15.8%)	18,399 (15.8%)	618 (15.4%)	

Of those undergoing recurrent emergent repairs, 3095 (77%) underwent one recurrent repair, 692 (17%) underwent two recurrent repairs, and 169 (4%) underwent three recurrent repairs. Among all beneficiaries undergoing recurrent emergent repair, 2015 (50%) experienced a 30-day complication and 1468 (36.5%) died throughout the study period.

### Survival analysis

**Fig. 1:**
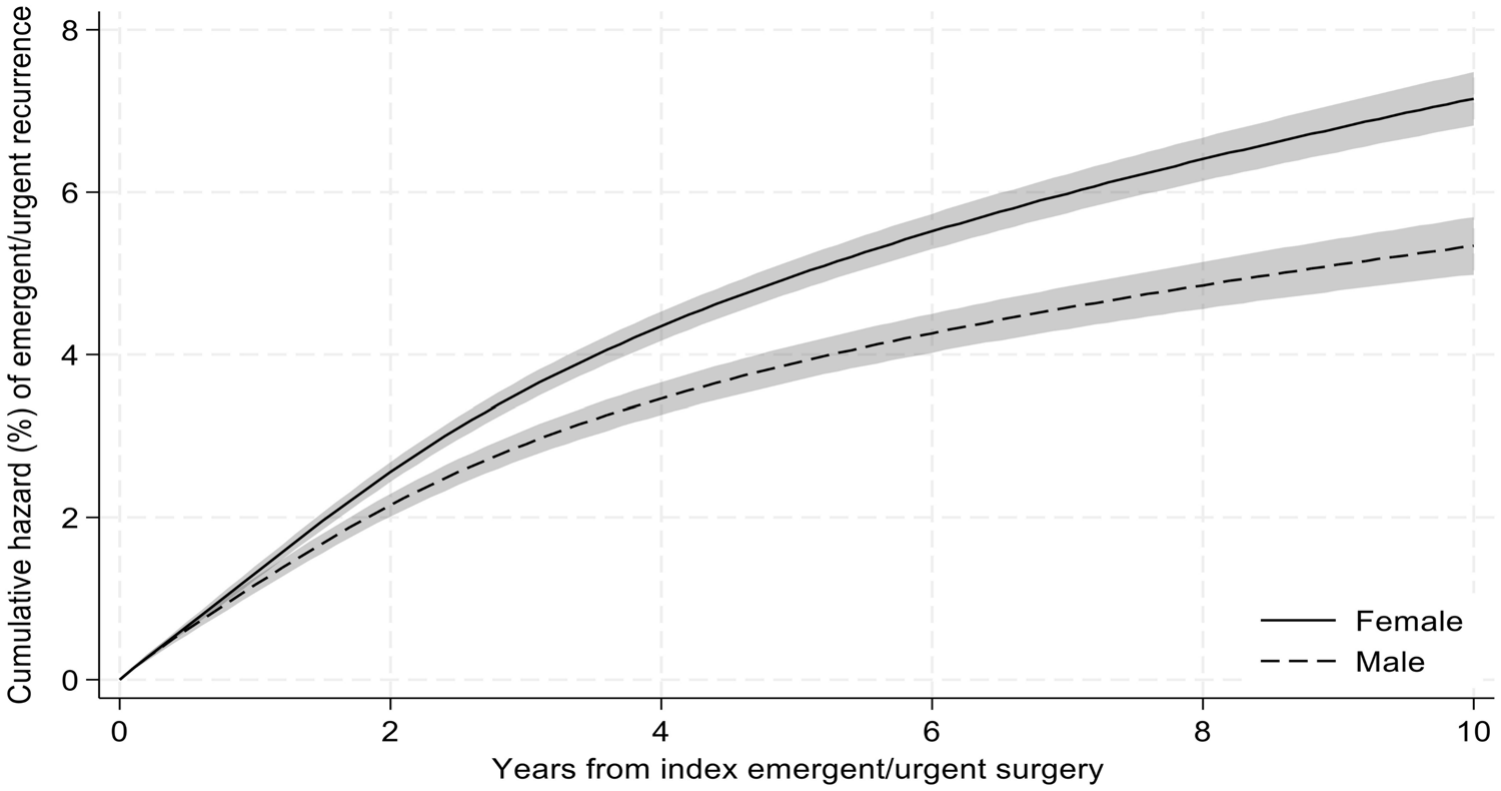
Cumulative hazard of recurrent emergent ventral hernia repair by sex. Cumulative recurrence of emergent/urgent ventral hernia repair was calculated using a flexible parametric survival hazards regression that adjusted for age, race/ethnicity, Elixhauser comorbidities, dual Medicare-Medicaid eligibility, beneficiary Medicare status code, surgical approach, hernia location, mesh use, component separation use, hernia surgeon volume, hospital bed size, teaching hospital status, hospital ownership, hospital nurse ratio, primary care health professional shortage designation, social vulnerability index, rural/urban area commuting status of beneficiary residence, and U.S. region of beneficiary residence. 95% confidence intervals represented by shaded area

**Fig. 2:**
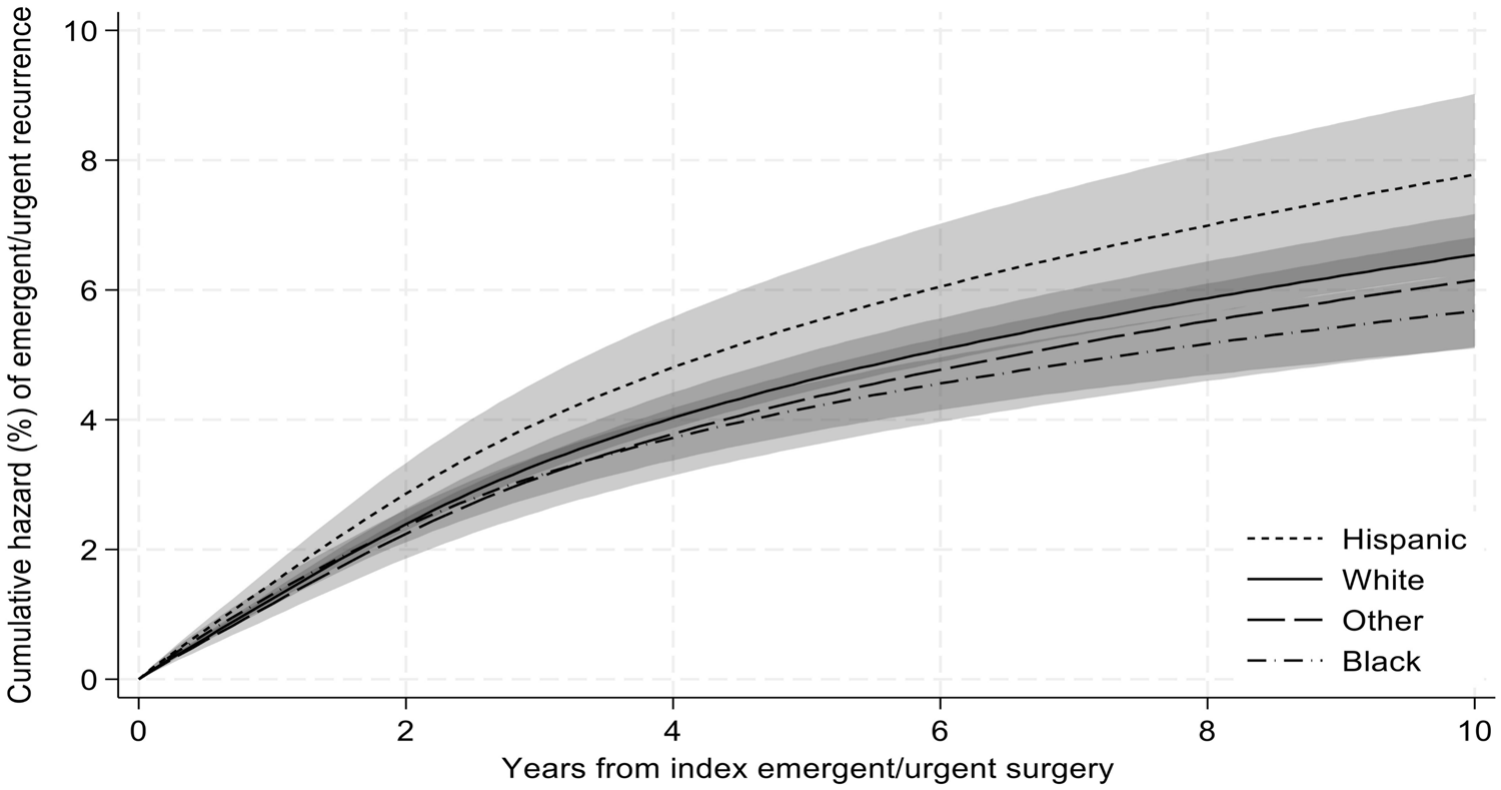
Cumulative hazard of recurrent emergent ventral hernia repair by race/ethnicity. Cumulative recurrence of emergent/urgent ventral hernia repair was calculated using a flexible parametric survival hazards regression that adjusted for age, sex, Elixhauser comorbidities, dual Medicare-Medicaid eligibility, beneficiary Medicare status code, surgical approach, hernia location, mesh use, component separation use, hernia surgeon volume, hospital bed size, teaching hospital status, hospital ownership, hospital nurse ratio, primary care health professional shortage designation, social vulnerability index, rural/urban area commuting status of beneficiary residence, and U.S. region of beneficiary residence. 95% confidence intervals represented by shaded area

At 10 years, the cumulative incidence of recurrent emergent hernia repair was 6.4% (95% CI 6.2–6.7). The median time to recurrent emergent repair was 1.6 years [IQR 0.8–3.2]. Risk-adjusted patient factors associated with increased risk for recurrent emergent repair include female sex (10-year Hazard Ratio [HR] 1.58, 95% CI [1.24–2.02], Fig. 1) and Hispanic ethnicity (10-year HR 1.19, 95% CI [1.01–1.41], Fig. 2). Compared to White beneficiaries, Black beneficiaries did not have an increased risk of emergent recurrent repair (10-year HR 0.75, 95% CI [0.53–1.05], Fig. 2). Dual-eligible beneficiaries similarly did not have any risk-adjusted difference in emergent recurrent repair (10-year HR 1.03, 95% CI [0.96–1.11]).

**Fig. 3:**
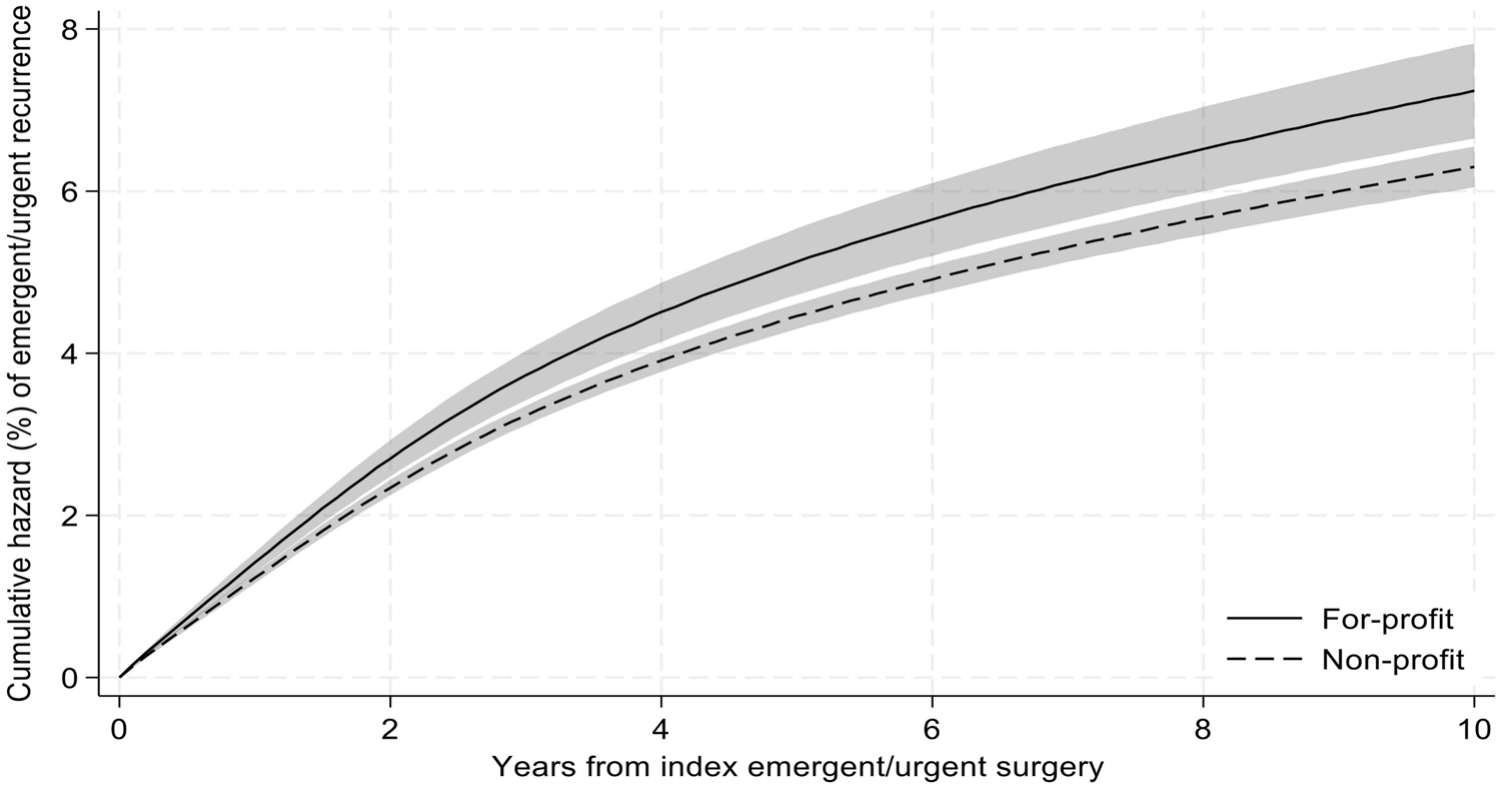
Cumulative hazard of recurrent emergent ventral hernia repair by hospital ownership. Cumulative recurrence of emergent/urgent ventral hernia repair was calculated using a flexible parametric survival hazards regression that adjusted for age, sex, race/ethnicity, Elixhauser comorbidities, dual Medicare-Medicaid eligibility, beneficiary Medicare status code, surgical approach, hernia location, mesh use, component separation use, hernia surgeon volume, hospital bed size, teaching hospital status, hospital nurse ratio, primary care health professional shortage designation, social vulnerability index, rural/urban area commuting status of beneficiary residence, and U.S. region of beneficiary residence. 95% confidence intervals represented by shaded area

For provider level factors, initial repairs done open demonstrated a higher risk for recurrent emergent repair (10-year HR 1.44, 95% CI [1.03- 2.01]) as compared to those done via minimally invasive (MIS) approaches. Additionally, beneficiaries with initial repairs performed at for-profit hospitals had higher risk for recurrent emergent repair (10-year HR 1.16, 95% CI [1.06–1.26], Fig. 3). Beneficiaries undergoing initial repair by surgeons that perform a high number of hernia repairs did not have reduced risk for undergoing a recurrent emergent repair (10-year HR 0.98, 95% CI [0.91–1.06]).

**Fig. 4:**
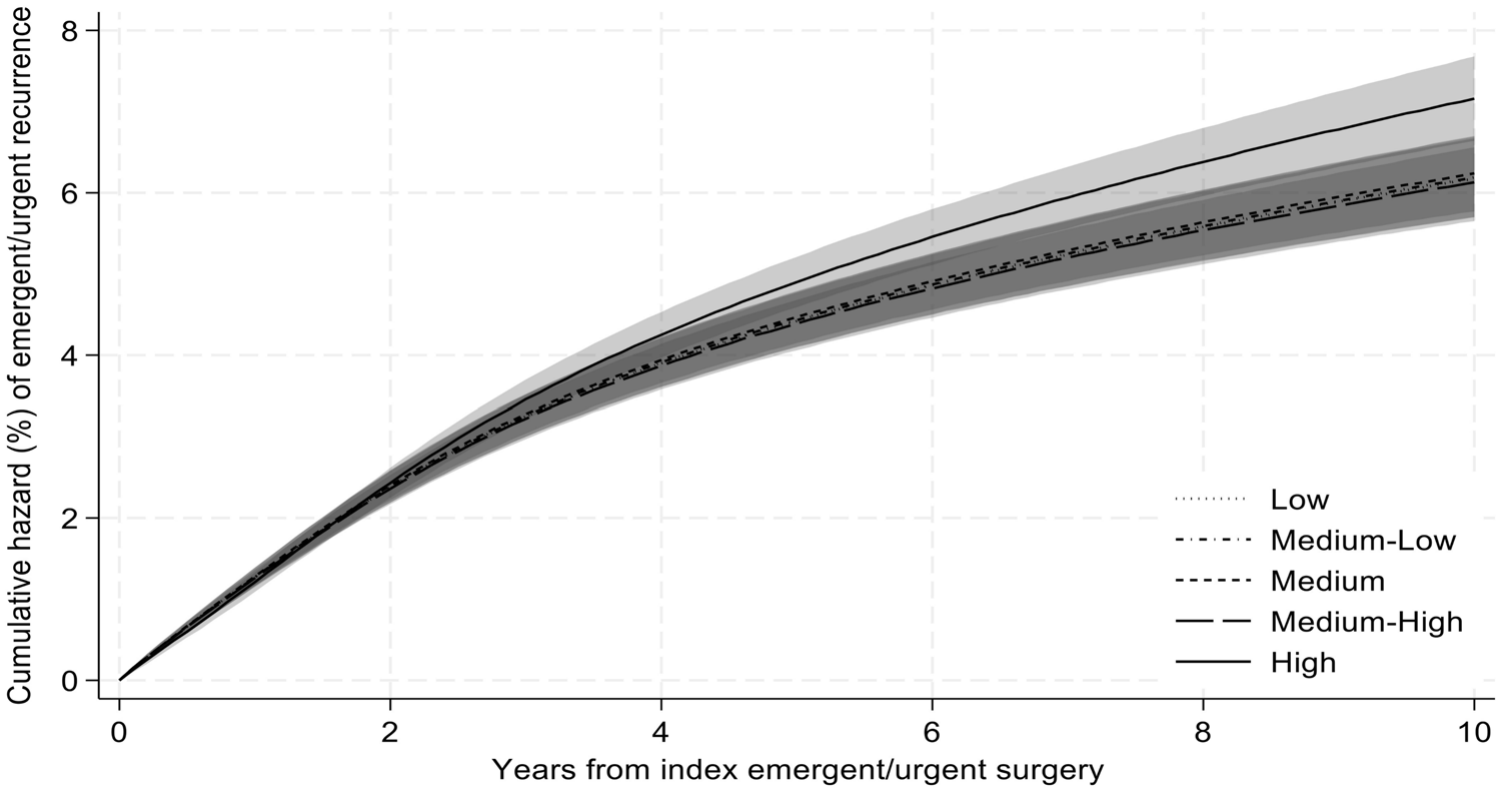
Cumulative hazard of recurrent emergent ventral hernia repair by social vulnerability. Cumulative recurrence of emergent/urgent ventral hernia repair was calculated using a flexible parametric survival hazards regression that adjusted for age, sex, race/ethnicity, Elixhauser comorbidities, dual Medicare-Medicaid eligibility, beneficiary Medicare status code, surgical approach, hernia location, mesh use, component separation use, hernia surgeon volume, hospital bed size, teaching hospital status, hospital ownership, hospital nurse ratio, primary care health professional shortage designation, rural/urban area commuting status of beneficiary residence, and U.S. region of beneficiary residence. 95% confidence intervals represented by shaded area

Patients in the highest quintile of social vulnerability had increased risk of undergoing recurrent emergent hernia repair compared to those in the lowest quintile (10-year HR 1.33, 95% CI [1.03–1.71], Fig. 4). In contrast, patients residing in whole county (10-year HR 1.00, 95% CI [0.89–1.11]) or partial county (10-year HR 1.00, 95% CI [0.89–1.13]) primary care HPSAs did not demonstrate any increased risk of recurrent emergent repair compared to those not living in a primary care HPSA.

## Discussion

Our nationally representative study on the risk factors associated with recurrent emergent hernia repairs among Medicare beneficiaries has three main findings. First, the 10-year cumulative incidence of undergoing recurrent emergent hernia repair was 6.4%. Second, female, Hispanic, or socially vulnerable patients are at highest risk of recurrent emergent repair. Lastly, initial repairs done open or at for-profit hospitals are associated with increased risk of recurrent emergent repair. Taken together, these findings provide insight into patient populations that may benefit from tailored care and opportunities for system-level intervention to improve outcomes for this common preference sensitive condition.

Prior literature has evaluated surgical disparities as it relates to “access-sensitive” conditions—i.e. diagnoses requiring surgery that preferably is planned, but when access is limited, may be delayed until worsening symptoms require riskier and costlier emergent surgery. For example, patients from minority populations, patients with higher social vulnerability, and patients from primary care HPSA’s are more susceptible to emergent operations and poor outcomes in these conditions [2, 19, 20]. In hernia care, existing literature has focused primarily on clinical risk factors that place patients at risk for initial emergent operations, such as age, comorbidities, and hernia characteristics [6, 7]. Studies of social factors are more limited, but have identified Black patients, patients on Medicaid, and socially vulnerable patients at increased risk as well [21, 22]. However, these studies were done in significantly smaller cohorts.

Our study extends these findings in several ways using a large nationally representative surgical cohort. First, we describe a group previously unexplored—patients that experience more than one emergent operation, arguably representing a population that exposes the widest vulnerabilities in our system. A 6.4% incidence of multiple emergent repairs is not insignificant and, given the prevalence of hernias, represents a notable opportunity for cost savings and prevention of harmful adverse outcomes for patients. Second, we evaluate the multidimensional risk factors that may be impacting this population, from a patient (demographic and socioeconomic), provider (surgeon volume, repair approach, hospital profit status), and community (rurality, SVI, HPSA) level. This contributes to the growing understanding of recognizing and addressing non-clinical factors that are driving health and surgical outcomes.

Opportunities for improvement should focus on strategies to address the at-risk populations we have identified and tailoring care for these patients. Hernia is unique in being preference sensitive, meaning surgery is not always recommended and is contingent on patient and surgeon decision making. This adds an additional layer of risk for vulnerable patients, as they may be susceptible to provider bias, lower engagement in shared decision making, lower health literacy, lower access to surgical expertise, and lower rates of meeting “optimization” cut offs that can lead to exclusion from surgery [3, 23–26]. However, an emergent operation has consistently worse outcomes than a “non-optimized” elective repair. For patients that may be at risk of recurrent emergent repairs and who are unlikely to have the resources for prehabilitation efforts (which have low success rates even in ideal circumstances [27, 28]), this presents an opportunity to make more nuanced decisions about repairing their hernia electively even in non-ideal settings. For example, much of the additional risk attributed to smoking or BMI can be mitigated by minimally invasive repairs, which is significantly more likely in an elective setting [29, 30]. As racial/ethnic minority patients have lower access to MIS surgeons, this is another potential intervention point [26]. Our study indicates that the use of MIS in the emergent setting, when feasible, could also help prevent additional need for emergent repairs.

From a systems level perspective, our study highlights a few key opportunities. First, despite a recent study finding that patients with access-sensitive surgical conditions (including hernia) have higher rates of emergent surgery in primary care HPSA’s, we did not see the same association in our study (nor an association with rural residence) [2]. This may indicate that the findings in that study were driven by the other included conditions, or that patients with multiple emergent operations face a more complex set of barriers aside from access to primary care. However, the association of for-profit hospital status and repeated emergent operations should prompt additional inquiry. One potential explanation for this is that for-profit hospitals tend to serve areas with higher unemployment and uninsured rates [31]. There may be unmeasured socioeconomic confounders contributing to this finding despite our risk-adjustment. However, there is a paucity of studies evaluating how hospital profit status may impact surgical care and further research is needed to understand this relationship.

Our study has several limitations. First, claims data may lack the clinical granularity needed to capture potential confounding factors influencing our outcomes, such as enterotomies, hernia size, the type of mesh used, the layer of the abdominal wall where the mesh was placed. These factors could influence the need for a subsequent emergent repair. However, a strength of this study is the population level analysis of risk after emergent hernia repair, among hernia specialists and general surgeons alike, that has limited the generalizability of previous studies. Additionally, we were unable to identify clinical recurrence without an operation, which would have provided a more direct comparison group. We performed an analysis comparing the emergent versus elective operative recurrences as a sensitivity check, and found that the characteristics of the elective repair group closely matched those of the no recurrent repair group, indicating these populations are similar. Lastly, the Medicare population may not be generalizable to all patients undergoing emergent hernia surgery. Reoperative recurrence rates may be lower than expected given the older patient population with an increased comorbidity burden. Despite this, hernia repair is more common in older adults both due to aging and the higher likelihood of developing an incisional hernia from a prior operation.

## Conclusion

Our study highlights multi-level risk factors associated with recurrent emergent hernia repairs, including patients that are female, Hispanic, and with high social vulnerability as well as patients receiving an open index repair and care at for-profit hospitals. This provides opportunities to intervene for populations that may benefit from different upfront approaches, closer follow-up, and earlier elective intervention for hernia recurrence.

## Supplementary Information

Below is the link to the electronic supplementary material.

Supplementary file1 (DOCX 315 KB)

Supplementary file2 (DOCX 31 KB)
