# Supplementary figures and images for: Recurrent emergent hernia repairs: who is at risk?
Source: Surg Endosc. 2025 Jun 19;39(7):4599–607. doi: 10.1007/s00464-025-11914-y (PMC12222345; doi:10.1007/s00464-025-11914-y)

Supplemental Table 1. ICD-9/10 and CPT codes used to identify index hernia repair operation


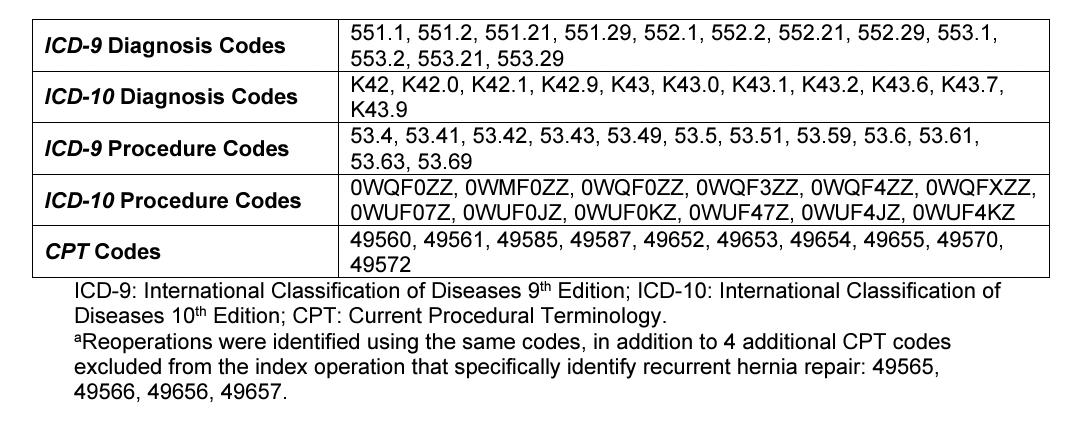

Supplement: Supplementary file 1 — Supplementary file1 (DOCX 315 KB) [file 464_2025_11914_MOESM1_ESM.docx]
