# Supplementary material for: Recurrent emergent hernia repairs: who is at risk?
Source: Surg Endosc. 2025 Jun 19;39(7):4599–607. doi: 10.1007/s00464-025-11914-y (PMC12222345; doi:10.1007/s00464-025-11914-y)
Supplement: Supplementary file 2 — Supplementary file2 (DOCX 31 KB) [file 464_2025_11914_MOESM2_ESM.docx]

Supplemental Table 2. Patient characteristics by recurrence status

| **Covariate** | | **Recurrence** | | | **p-value^a^** |  |
| --- | --- | --- | --- | --- | --- | --- |
|  |  |  |  |  |  |  |
|  |  | **No recurrent operation**  **(n=108099) % column** | **Elective**  **(n=8110)**  **% column** | **Emergent/urgent (n=4018) % column** |  |  |
| Age | mean (SD) | 71.9 (11.9) | 66.9 (11.6) | 67.3 (12.5) | 0.042 |  |
| Sex | Female | 62624 (57.9%) | 4753 (58.6%) | 2695 (67.1%) | <0.001 |  |
|  | Male | 45475 (42.1%) | 3357 (41.4%) | 1323 (32.9%) |  |  |
| Race | White | 88962 (82.3%) | 6905 (85.1%) | 3179 (79.1%) | <0.001 |  |
|  | Black | 12672 (11.7%) | 743 (9.2%) | 558 (13.9%) |  |  |
|  | Hispanic | 2677 (2.5%) | 201 (2.5%) | 149 (3.7%) |  |  |
|  | Other Races | 3788 (3.5%) | 261 (3.2%) | 132 (3.3%) |  |  |
| Repair Approach | Open | 90582 (83.8%) | 6957 (85.8%) | 3437 (85.5%) | 0.719 |  |
|  | MIS | 17517 (16.2%) | 1153 (14.2%) | 581 (14.5%) |  |  |
| Repair Location | Incisional-ventral | 71606 (66.2%) | 5951 (73.4%) | 3201 (79.7%) | <0.001 |  |
|  | Umbilical | 36493 (33.8%) | 2159 (26.6%) | 817 (20.3%) |  |  |
| Mesh Use | | 44827 (41.5%) | 3080 (38.0%) | 1709 (42.5%) | <0.001 |  |
| Component Separation Use | | 4639 (4.3%) | 277 (3.4%) | 170 (4.2%) | 0.025 |  |
| Number of Elixhauser comorbidities (out of 29) | Zero | 4684 (4.3%) | 502 (6.2%) | 162 (4.0%) | <0.001 |  |
|  | One | 11997 (11.1%) | 1187 (14.6%) | 426 (10.6%) |  |  |
|  | Two and above | 91418 (84.6%) | 6421 (79.2%) | 3430 (85.4%) |  |  |
| Hernia Surgeon Volume [Mean: Low=13.8, Med=39.4, High=110.2] | Low (1 to 25) | 36518 (33.8%) | 2558 (31.5%) | 1326 (33.0%) | 0.073 |  |
|  | Medium (26 to 56) | 35695 (33.0%) | 2759 (34.0%) | 1389 (34.6%) |  |  |
|  | High (57+) | 35886 (33.2%) | 2793 (34.4%) | 1303 (32.4%) |  |  |
| Dual (Medicare-Medicaid) Eligible | | 30024 (27.8%) | 2261 (27.9%) | 1407 (35.0%) | <0.001 |  |
| Beneficiary Medicare Status Code | Aged with ESRD | 2487 (2.3%) | 127 (1.6%) | 102 (2.5%) | 0.001 |  |
|  | Aged without end-stage renal disease (ESRD) | 84756 (78.4%) | 5568 (68.7%) | 2654 (66.1%) |  |  |
|  | Disabled with ESRD | 1949 (1.8%) | 213 (2.6%) | 107 (2.7%) |  |  |
|  | Disabled without ESRD | 17685 (16.4%) | 2063 (25.4%) | 1087 (27.1%) |  |  |
|  | ESRD only | 1222 (1.1%) | 139 (1.7%) | 68 (1.7%) |  |  |
| Hospital Bed size | <250 | 42143 (39.0%) | 3243 (40.0%) | 1521 (37.9%) | 0.055 |  |
|  | 250-499 | 37991 (35.1%) | 2807 (34.6%) | 1415 (35.2%) |  |  |
|  | 500+ | 27965 (25.9%) | 2060 (25.4%) | 1082 (26.9%) |  |  |
| Teaching Hospital | non-Teaching | 34966 (32.4%) | 2978 (36.7%) | 1359 (33.8%) | 0.002 |  |
|  | Teaching | 73133 (67.7%) | 5132 (63.3%) | 2659 (66.2%) |  |  |
| Hospital Ownership | For-profit | 15517 (14.4%) | 1229 (15.2%) | 645 (16.1%) | 0.432 |  |
|  | Not-for-profit | 81048 (75.0%) | 5974 (73.7%) | 2925 (72.8%) |  |  |
|  | Other (government) | 11534 (10.7%) | 907 (11.2%) | 448 (11.2%) |  |  |
| Nurse Staffing Ratio | Mean (SD) | 8.69 (3.41) | 8.63 (3.28) | 8.57 (13.67) | 0.731 |  |
| Health Professional Shortage Area (HPSA) | Non-HPSA | 11263 (10.4%) | 929 (11.5%) | 422 (10.5%) | 0.291 |  |
|  | Part County | 74946 (69.3%) | 5201 (64.1%) | 2607 (64.9%) |  |  |
|  | Whole County | 21890 (20.3%) | 1980 (24.4%) | 989 (24.6%) |  |  |
| Social Vulnerability Index | Low | 18297 (16.9%) | 1409 (17.4%) | 611 (15.2%) | <0.001 |  |
|  | Medium-Low | 19679 (18.2%) | 1497 (18.5%) | 677 (16.9%) |  |  |
|  | Medium | 20621 (19.1%) | 1632 (20.1%) | 742 (18.5%) |  |  |
|  | Medium-High | 23129 (21.4%) | 1746 (21.5%) | 850 (21.2%) |  |  |
|  | High | 26373 (24.4%) | 1826 (22.5%) | 1138 (28.3%) |  |  |
| Population Density, Urbanization, and Daily Commuting | Not-Rural | 85033 (78.7%) | 6247 (77.0%) | 3192 (79.4%) | 0.003 |  |
|  | Rural | 23066 (21.3%) | 1863 (23.0%) | 826 (20.6%) |  |  |
| U.S. Region | South | 42965 (39.8%) | 3297 (40.7%) | 1618 (40.3%) | 0.003 |  |
|  | Midwest | 25710 (23.8%) | 1940 (23.9%) | 893 (22.2%) |  |  |
|  | Northeast | 22322 (20.7%) | 1576 (19.4%) | 889 (22.1%) |  |  |
|  | West | 17102 (15.8%) | 1297 (16.0%) | 618 (15.4%) |  |  |

1. *p-value for the comparison of the patient characteristics between those having elective vs. emergent/urgent recurrences
